# Association of Autoimmune Addison's Disease with Alleles of STAT4 and GATA3 in European Cohorts

**DOI:** 10.1371/journal.pone.0088991

**Published:** 2014-03-10

**Authors:** Anna L. Mitchell, Katie D. R. Macarthur, Earn H. Gan, Lucy E. Baggott, Anette S. B. Wolff, Beate Skinningsrud, Hazel Platt, Andrea Short, Anna Lobell, Olle Kämpe, Sophie Bensing, Corrado Betterle, Anna Kasperlik-Zaluska, Magdalena Zurawek, Marta Fichna, Ingrid Kockum, Gabriel Nordling Eriksson, Olov Ekwall, Jeanette Wahlberg, Per Dahlqvist, Anna-Lena Hulting, Marissa Penna-Martinez, Gesine Meyer, Heinrich Kahles, Klaus Badenhoop, Stephanie Hahner, Marcus Quinkler, Alberto Falorni, Amanda Phipps-Green, Tony R. Merriman, William Ollier, Heather J. Cordell, Dag Undlien, Barbara Czarnocka, Eystein Husebye, Simon H. S. Pearce

**Affiliations:** 1 Institute of Genetic Medicine, Newcastle University, Newcastle, United Kingdom; 2 Department of Clinical Science, University of Bergen, Bergen, Norway; 3 Department of Medical Genetics, Oslo University Hospital, Oslo, Norway; 4 University of Oslo, Oslo, Norway; 5 CIGMR, Institute of Population Health, University of Manchester, Manchester, United Kingdom; 6 Uppsala University, Uppsala, Sweden; 7 Karolinska Institutet, Stockholm, Sweden; 8 Department of Medicine, University of Padua School of Medicine, Padua, Italy; 9 Department of Endocrinology, Center for Postgraduate Medical Education, Warsaw, Poland; 10 Institute of Human Genetics, Polish Academy of Sciences, Poznan, Poland; 11 Neuroimmunology Unit, Department of Clinical Neuroscience and Centrum for Molecular Medicine, Karolinska Institutet, Stockholm, Sweden; 12 Department of Molecular Medicine and Surgery, Karolinska Institutet, Stockholm, Sweden; 13 The Sahlgrenska Academy, University of Gothenburg, Gothenburg, Sweden; 14 Department of Medical and Health Sciences, Division of Endocrinology, Faculty of Health Sciences, Linköping University, Linköping, Sweden; 15 Department of Public Health and Clinical Medicine, Umeå University, Umeå, Sweden; 16 Department Medicine (Division Endocrinology), University Hospital Frankfurt, Goethe-University, Frankfurt, Germany; 17 University Hospital Wuerzburg (Department of Medicine, Endocrinology and Diabetology), Wuerzburg, Germany; 18 Clinical Endocrinology, Charité Campus Mitte, Charité University Medicine Berlin, Berlin, Germany; 19 Department of Internal Medicine, University of Perugia, Perugia, Italy; 20 Department of Biochemistry, University of Otago, Otago, New Zealand; 21 Department of Biochemistry and Molecular Biology, Center for Postgraduate Medical Education, Warsaw, Poland; 22 Department of Medicine, Haukeland University Hospital, Bergen, Norway; University of Siena, Italy

## Abstract

**Background:**

Gene variants known to contribute to Autoimmune Addison's disease (AAD) susceptibility include those at the MHC, MICA, CIITA, CTLA4, PTPN22, CYP27B1, NLRP-1 and CD274 loci. The majority of the genetic component to disease susceptibility has yet to be accounted for.

**Aim:**

To investigate the role of 19 candidate genes in AAD susceptibility in six European case-control cohorts.

**Methods:**

A sequential association study design was employed with genotyping using Sequenom iPlex technology. In phase one, 85 SNPs in 19 genes were genotyped in UK and Norwegian AAD cohorts (691 AAD, 715 controls). In phase two, 21 SNPs in 11 genes were genotyped in German, Swedish, Italian and Polish cohorts (1264 AAD, 1221 controls). In phase three, to explore association of GATA3 polymorphisms with AAD and to determine if this association extended to other autoimmune conditions, 15 SNPs in GATA3 were studied in UK and Norwegian AAD cohorts, 1195 type 1 diabetes patients from Norway, 650 rheumatoid arthritis patients from New Zealand and in 283 UK Graves' disease patients. Meta-analysis was used to compare genotype frequencies between the participating centres, allowing for heterogeneity.

**Results:**

We report significant association with alleles of two STAT4 markers in AAD cohorts (rs4274624: P = 0.00016; rs10931481: P = 0.0007). In addition, nominal association of AAD with alleles at GATA3 was found in 3 patient cohorts and supported by meta-analysis. Association of AAD with CYP27B1 alleles was also confirmed, which replicates previous published data. Finally, nominal association was found at SNPs in both the NF-κB1 and IL23A genes in the UK and Italian cohorts respectively.

**Conclusions:**

Variants in the STAT4 gene, previously associated with other autoimmune conditions, confer susceptibility to AAD. Additionally, we report association of GATA3 variants with AAD: this adds to the recent report of association of GATA3 variants with rheumatoid arthritis.

## Introduction

Autoimmune Addison's disease (AAD) is a rare autoimmune endocrinopathy with a prevalence of 110–140 cases per million in Caucasian European populations [1], [2]. Like many autoimmune endocrine conditions, AAD has a strong and oligo-genetic basis. The first case report of monozygotic twins concordant for AAD, suggesting a genetic aetiology, was published more than 40 years ago [3] and a number of similar cases have since been reported [4]–[6]. The observation that AAD, in common with many other autoimmune conditions, sometimes clusters within families also supports a genetic basis for the condition [7], [8]. Furthermore, individuals with AAD are predisposed to develop other organ-specific autoimmune diseases which suggests shared susceptibility loci for these conditions. Most cases of AAD are not attributable to Mendelian abnormalities, but are a complex genetic trait, whereby currently unknown environmental factors interact with a number of genetic variants to cause disease.

The hypothesis-driven candidate gene approach has been used to investigate numerous complex diseases to date. In this method, plausible candidates are selected for investigation based on what is known of the biology and pathophysiology of a disease, on known allelic associations with mechanistically-related diseases and from information gained from the investigation of monogenic forms of a condition. In AAD such a monogenic form exists, as the autoimmune polyendocrinopathy syndrome type 1 (APS1), caused by loss of function mutations to both alleles of the *AIRE* gene [9]. In the investigation of complex AAD, the candidate gene approach has seen a number of successes, most notably the finding, and replication, of association of *MHC* alleles [10]–[13] and polymorphisms in *PTPN22* [14], [15] and *CTLA4* [16], [17] with AAD, all of which were investigated after having been associated with related autoimmune conditions. In AAD, previous candidate gene studies have been relatively small and some findings have proved difficult to replicate [18], [19]. Therefore, in order to attain adequate power in candidate gene studies, large case-control cohorts are needed. The EURADRENAL consortium has recently provided a platform for collaboration between researchers in Europe and has, for the first time, allowed a large number of AAD DNA samples to be aggregated for genetic analysis.

This study aimed to investigate the role of 19 candidate genes in the pathogenesis of AAD in six European case-control cohorts using the Sequenom iPlex genotyping platform.

## Subjects and Methods

Ethical approval for this work was obtained in each participating country as follows: Padua, Italy - Regione del Veneto Azienda Ospedaliera di Padova (ref 1583P); Perugia, Italy - CEAS Umbria (1247/08); Poznan, Poland - Ethics Committee at the Poznan University of Medical Sciences (18.06.2009; decision # 540/09); Warsaw, Poland - Ethical committee at the Center of Postgraduate Medical Education (April 18, 2007); Sweden - Regionala etikprövningsnämnden i Stockholm Dnr 2008/296-31/2; Oslo, Norway – Oslo Regional Ethics committee; Bergen, Norway - Regional Ethics Committee West; Newcastle, UK - Leeds (East) Research Ethics Committee, 2005 (REC reference number 05/Q1206/144); Frankfurt, Germany - Ethical committee of the University Hospital, Goethe-University, Frankfurt am Main (Reference-No 49/09); New Zealand - The New Zealand Multi-Region Ethics Committee (reference OTA/99/02/007).

Informed, written consent was sought from each study participant at all centres with the exception of the Norwegian controls. These samples were gathered through the national blood donor scheme. All blood donors are informed of ongoing research through written information and are given the opportunity to opt out should they wish to do so. All samples collected in this way are anonymised at source.

In each subject with AAD, the clinical diagnosis was confirmed by either a low basal cortisol with a high ACTH level or a subnormal response to the short synacthen test (250 µg parenteral synthetic ACTH_1–24_). Patients with primary adrenal failure due to adrenal gland infiltration or infection, with secondary adrenal failure, or with APS1 (on the grounds of mucocutaneous candidiasis, hypoparathyroidism, and/or ectodermal dystrophy) were excluded. In total, DNA samples from 1,955 individuals with AAD and 1,936 healthy controls from 6 European countries were available for analysis. Available cohort characteristics are shown in table 1. 21-hydroxylase (21OH) autoantibody status was not available for all AAD cases included in this study, as it is not routinely tested in all participating countries. In total, 1,204 cases (61.6% of the total cohort) were known to be 21OH autoantibody positive (21OH+): 53 from UK, 290 from Norway, 73 from Poland, 154 from Germany, 266 from Italy and 368 from Sweden. All control samples included were Caucasian and had no personal or family history of autoimmune disease. Clinically silent autoimmune disease was not excluded in these controls by checking autoantibody levels, adrenal or thyroid function, however 21OH positivity in controls is known to be very rare.

**Table 1:** Cohort information.

AAD cohort	Cohort size (cases/controls)	Mean age of onset (range)	Male∶Female	Additional autoimmune comorbidities*		
				All (% of cohort)	Autoimmune thyroid disease (%)	Type 1 diabetes (%)
**UK**	309/335	39 (10–83 years)	1∶3.3	57	43	6
**Norway**	382/380	53 (18–95 years)	1∶1.7	66	47	12
**Germany**	341/235	51 (22–88 years)	1∶1.3	55	47	7
**Italy**	280/322	38 (6–84 years)	1∶2.0	72	61	12
**Poland**	275/296	38 (9–76 years)	1∶3.0	77	38	10
**Sweden**	368/368	34 (0–71 years)	1∶1.6	62	49	11

Information for each of the six included AAD cohorts.

*Additional autoimmune comorbidities include type 1 diabetes, autoimmune thyroid disease (Graves' and autoimmune hypothyroidism), pernicious anaemia, vitiligo, autoimmune hepatitis, rheumatoid arthritis, SLE, Sjogren's disease, Coeliac disease, premature ovarian failure, alopecia.

Members of the EURADRENAL consortium selected 19 candidate genes for investigation, based on current knowledge of immunological pathways and the aetiology of autoimmune conditions. These included genes influencing CD4^+^ lymphocyte fate (*GATA3, GATA binding protein 3; IL17A, interleukin 17A; IL17RA, interleukin 17 receptor A; IL21, interleukin 21; IL23A, interleukin 23 alpha subunit p19; RORA, RAR-related orphan receptor A; RORC, RAR-related orphan receptor C; STAT2, signal transducer and activator of transcription 2; STAT4, signal transducer and activator of transcription 4* and *TBX21, T-box 21*), transcription factors which alter the immune response (*NFATC2, nuclear factor of activated T-cells, cytoplasmic, calcineurin-dependent 2, NFKB1, nuclear factor of kappa light polypeptide gene enhancer in B-cells 1* and *REL, v-rel reticuloendotheliosis viral oncogene homolog*) and those genes important for innate immune mechanisms (*CYP2R1, vitamin D 25-hydroxylase; CYP24A1, 1,25-dihydroxyvitamin D3 24-hydroxylase; CYP27B1, 25-hydroxyvitamin D-1 alpha hydroxylase; GC, vitamin D binding protein; IFIH1, interferon induced with helicase C domain 1* and *VDR, vitamin D receptor*).

SNPs in these candidate genes were selected for genotyping using the HapMap database tag-SNP picker (www.Hapmap.org) [20]. SNPs were chosen with consideration of linkage disequilibrium (LD) patterns in CEU subjects which were studied in Haploview [21], to ensure that the major haplotypes across each gene were represented in the data collected, as far as possible. Independent SNPs (those with an r^2^ of less than 0.4) with a minor allele frequency (MAF) of greater than 0.1 were preferentially selected for genotyping.

In this study, all SNP genotyping was carried out using Sequenom MassARRAY technology (Sequenom, San Diego) at either CIGMR, Manchester University, UK or NewGene, Newcastle University, UK. PCR reactions were set up in a 10 µl volume and contained 30 ng of template DNA, 1.25× PCR buffer, 1 mM MgCl_2_, 500 µM dNTPs and 0.5 U/reaction of Fast Start Taq polymerase (Roche). A pool of primers (Metabion) was made to give a final concentration of each primer of 100 nM. Primer sequences are available from the authors on request. The thermal cycling conditions for the reaction included an initial denaturation step at 94°C for 15 minutes, followed by 35 cycles of 94°C for 20 seconds, 56°C for 30 seconds and 72°C for 1 minute, followed by a final extension step of 72°C for 3 minutes. The iPlex™ assay was then followed according to manufacturer's instructions (http://www.sequenom.com).

Genotyping was undertaken in three phases, with a statistical analysis performed after each phase. In the first phase, 85 tag SNPs in and around the 19 chosen candidate genes were genotyped in the UK (309 AAD, 335 controls) and Norwegian (382 cases, 380 controls) cohorts. Genes associated with one or both cohorts in this analysis were then selected for replication in phase 2 of this study, where 21 SNPs in 11 genes were genotyped in AAD case and control cohorts from Germany (341 AAD, 235 controls), Poland (275 AAD, 296 controls), Italy (280 AAD, 322 controls) and Sweden (368 AAD, 368 controls). Finally, in phase 3 of the study, 15 SNPs in the *GATA3* gene were genotyped in AAD cases and controls from the UK (335 AAD, 302 controls) and from Norway (352 AAD, 1,353 controls). In addition, to determine whether the association with *GATA3* polymorphisms extended to other autoimmune conditions, these SNPs were also genotyped in a cohort of 1,195 type 1 diabetes patients and matched controls from Norway, in 650 rheumatoid arthritis patients from New Zealand and 452 ancestrally-matched controls and in a cohort of 283 UK Graves' disease patients.

### Data management, Quality Control and Statistical analysis

Genotyping call rates were first calculated (AA+Aa+aa/sample number ×100) and any SNP with a call rate of less than 95% was excluded from further analysis. Control genotype data were used to check for Hardy-Weinberg equilibrium (HWE). SNPs out of HWE (P<0.01) in the control population were excluded from further analysis. The prevalence of genotypes (AA vs Aa vs aa) and alleles (A = 2xAA+Aa, a = 2xaa+Aa) was calculated for all SNPs. χ^2^ testing on 2×2 and 3×2 contingency tables was used to analyse the data for association. To account for multiple testing, a Bonferroni correction was applied (0.05/number of independent loci tested). Independent loci were defined as those with an r^2^ value, derived from Hapmap CEU data, of less than 0.4.

A meta-analysis, using the Review Manager (RevMan) Version 5.0 program (The Nordic Cochrane Centre, Copenhagen, Denmark [22]), was then undertaken, using a random effects model to calculate odds ratios, confidence intervals and two-sided p-values. The impact of heterogeneity between the cohorts was estimated using an *I^2^* index. Results are stated as P-values.

## Results

### Phase 1

#### Phase 1 UK cohort results

In total, alleles of 13 SNPs in 9 genes showed nominal association (P<0.05) with AAD in the UK cohort (table 2). Maximal association was seen with the *NF-κB1* gene. Six SNPs were genotyped in, and close to, this gene. Alleles at 3 SNPs in moderate LD (r^2^ 0.39–0.68), *rs10026278*, *rs230532* and *rs4698861*, were associated with AAD in the UK cases compared to controls. Strongest evidence for association was at *rs4698861*, where the frequency of the minor (G) allele was 27.4% in AAD cases vs 37.4% in controls [odds ratio (OR) 0.63, 95% confidence interval (CI) 0.50–0.80; P = 0.00017)]. Haplotype analysis in UNPHASED [23] revealed that the marker *rs4698861* accounts for all of the association with disease: if conditioned upon, no association with other markers in close proximity is seen. Nominal allelic association was also found at markers in *CYP24A1*, *CYP27B1, GATA3, IL17A, IL21, IL23A, REL* and *STAT2* (table 2). Allowing for 54 independent loci tested (P 0.05/54 = ≤0.00096), two of the above associations, both in the *NF-κB1* gene (*rs230532* P_allele_ = 0.00041; *rs4698861* P_allele_ = 0.00017) meet the threshold for significance. Full genotype results for all cohorts can be found in File S1.

**Table 2:** Associations with AAD in the UK and Norwegian cohorts in phase 1 of genotyping.

	Gene	SNPs typed	SNPs excluded	rs ID	Minor allele	MAF cases/controls	P_genotype_/P_allele_	OR [95% CI]	LD between associated markers*
UK	**NFKB1**	6	0	rs10026278	T	0.27/0.35	**0.012/0.0034**	0.69 [0.54–0.88]	Moderate
				rs230532	T	0.30/0.40	**0.0016/0.00041**	0.65 [0.52–0.82]	
				rs4698861	G	0.27/0.37	**0.00084/0.00017**	0.63 [0.50–0.80]	
	**CYP27B1**	3	1	rs4646536	G	0.26/0.33	**0.012/0.0091**	0.72 [0.56–0.92]	Significant
				rs703842	G	0.27/0.33	**0.027/0.014**	0.74 [0.58–0.94]	
	**IL23A**	1	0	rs11170816	A	0.05/0.09	N/A/**0.0047**	0.53 [0.34–0.84]	
	**REL**	2	1	rs13017599	A	0.41/0.33	**0.0099/0.0028**	1.40 [1.12–1.76]	
	**GATA3**	4	0	rs569421	C	0.26/0.19	**0.0092/0.003**	1.50 [1.15–1.96]	Low
				rs444929	C	0.21/0.28	**0.012/0.0053**	0.69 [0.54–0.90]	
	**IL21**	2	1	rs907715	T	0.32/0.39	**0.015/0.012**	0.74 [0.59–0.93]	
	**STAT2**	2	1	rs2066808	G	0.05/0.09	**0.039/0.012**	0.57 [0.36–0.90]	
	**CYP24A1**	9	3	rs4809959	G	0.48/0.53	**0.012/0.046**	0.80 [0.64–0.99]	
	**IL17A**	3	0	rs16882180	T	0.32/0.38	0.13/**0.043**	0.79 [0.63–1.00]	
*Norway*	***STAT4***	*11*	*2*	*rs4274624*	*C*	*0.27/0.19*	***0.0013/0.00045***	*1.52 [1.20–1.94]*	
	***RORA***	*4*	*1*	*rs1234805*	*T*	*0.37/0.30*	***0.0068/0.0018***	*1.41 [1.13–1.75]*	
	***GATA3***	*4*	*0*	*rs3802604*	*G*	*0.32/0.37*	***0.04/0.038***	*0.79 [0.64–0.98]*	
	***NFKB1***	*6*	*2*	*rs228611*	*A*	*0.43/0.49*	*0.063/* ***0.024***	*0.79 [0.65–0.97]*	
	***CYP24A1***	*9*	*1*	*rs2209314*	*C*	*0.25/0.25*	***0.031/0.91***	*0.99 [0.78–1.25]*	
	***IL17A***	*3*	*1*	*rs4711998*	*A*	*0.22/0.27*	*0.060/* ***0.039***	*0.77 [0.61–0.98]*	

Nominally significant associations with AAD in the UK and Norwegian (italic text) cohorts in phase 1 of genotyping - no association was observed with alleles at *NFATC2, RORC, TBX21, CYP2R1, GC, IFIH1, IL17RA* and *VDR* (data not shown). P_genotype_ and P_allele_ denote the P values derived from 2×3 and 2×2 chi squared testing respectively.

*Low LD = r^2^<0.40, moderate LD = r^2^ 0.40–0.79, significant LD = r^2^>0.79.

If the minor genotype was not represented in the dataset, the P_genotype_ result is recorded as N/A.

#### Phase 1 Norwegian cohort results

In total, 6 SNPs in 6 genes were associated with AAD in the Norwegian cohort (table 2). Maximal association was seen at *rs4274624* in the *STAT4* gene. In total, 9 SNPs were genotyped at this locus, but in this cohort, 2 were excluded (*rs10931481* and *rs4853543*) due to low genotyping call rates. Of the remaining 7 SNPs, only alleles at *rs4274624* were associated with AAD, with the minor (C) allele frequency being 26.9% in cases and 19.5% in controls (P = 0.00045, OR 1.52, [95% CI 1.20–1.94]). SNPs in an additional 5 genes, *IL17A, CYP24A1, GATA3, NFKB1* and *RORA* showed nominal significance. However, accounting for multiple comparisons, only *rs4274624* in *STAT4* remained significantly associated (P = 0.00045) in the Norwegian cohort.

### Phase 2

#### Phase 2 European cohort results

Analysis of genotype data for each of the six different European control cohorts using χ^2^ testing indicated significant genetic heterogeneity between the populations studied (table 3), with the highest levels of heterogeneity between control cohorts from Italy and the UK and the least heterogeneity between the German and Swedish cohorts.

**Table 3:** Genetic heterogeneity between the six different European control cohorts.

	UK	Norway	Germany	Italy	Poland	Sweden
UK		16.7%	21.4%	73.3%	53.3%	46.7%
Norway			7.7%	64.3%	30.8%	14.3%
Germany				50.0%	5.3%	0.0%
Italy					60.0%	57.1%
Poland						20.0%
Sweden						

Genetic heterogeneity between the six different European control cohorts. Allele frequencies were compared between all control populations using a chi squared test. The percentage of comparisons where a statistically significant result was seen (P<0.05) is shown in the table. A low percentage indicates that there were few markers at which allele frequencies differed between 2 populations of interest suggesting that there is little genetic heterogeneity between those two populations. A high percentage indicates that allele frequencies between two populations differed significantly at multiple markers, suggesting significant genetic heterogeneity between those two populations. Between the German and Swedish control cohorts, allele frequencies did not differ significantly at a single marker, suggesting that these populations are relatively genetically similar. By contrast, the UK and Italian control cohorts differed significantly at 73% of markers tested, suggesting significant genetic heterogeneity between these two cohorts.

The phase 2 results for each population are summarised in table 4. Allowing for correction in the testing of 15 independent loci in the individual cohorts (P 0.05/15 = ≤0.0033), alleles at 1 marker only were significantly associated with AAD in the Italian cohort (*rs11171806* in *IL23A*; P = 0.0028).

**Table 4:** Associations with AAD in the German, Swedish, Italian and Polish cohorts in phase 2 of genotyping.

Gene	SNPs typed	SNPs excluded	rs ID	Minor allele	MAF cases/controls	P_genotype_/P_allele_	OR [95% CI]	LD between associated markers*
**Germany**								
**IL21**	2	0	rs907715	T	0.24/0.31	**0.0078/0.018**	0.73 [0.56–0.95]	
**Sweden**								
**STAT4**	3	0	rs4274624	C	0.29/0.24	0.056/**0.017**	1.33 [1.10–1.68]	
**Italy**								
**STAT4**	3	0	rs10931481	G	0.36/0.29	**0.016/0.0056**	1.41 [1.11–1.80]	Moderate
			rs4274624	C	0.28/0.22	0.059/**0.02**	1.37 [1.10–1.78]	
**IL23A**	1	0	rs11171806	A	0.06/0.03	**0.012/0.0028**	2.37 [1.32–4.23]	
**NFKB1**	3	0	rs10026278	T	0.27/0.23	**0.049**/0.078	1.27 [0.97–1.65]	
**STAT2**	2	0	rs2066808	G	0.07/0.04	**0.037/0.014**	1.93 [1.13–3.28]	Significant
			rs2066807	G	0.06/0.03	**0.023/0.0063**	2.18 [1.23–3.85]	
**Poland**								
**IL21**	2	0	rs2221903	C	0.31/0.38	**0.04/0.02**	0.75 [0.58–0.96]	
**CYP24A1**	1	0	rs4809959	G	0.56/0.49	0.11/**0.03**	1.29 [1.02–1.64]	
**GATA3**	3	0	rs444929	C	0.18/0.21	**0.03**/0.12	0.79 [0.59–1.06]	

Nominally significant associations with AAD in the German, Swedish, Italian and Polish cohorts in phase 2 of genotyping. No association was observed with alleles at *RORA, IL17A, CYP27B1* and *REL* (data not shown).

*Low LD = r^2^<0.40, moderate LD = r^2^ 0.40–0.79, significant LD = r^2^>0.7.

#### Meta-analysis

**Figure 1:**
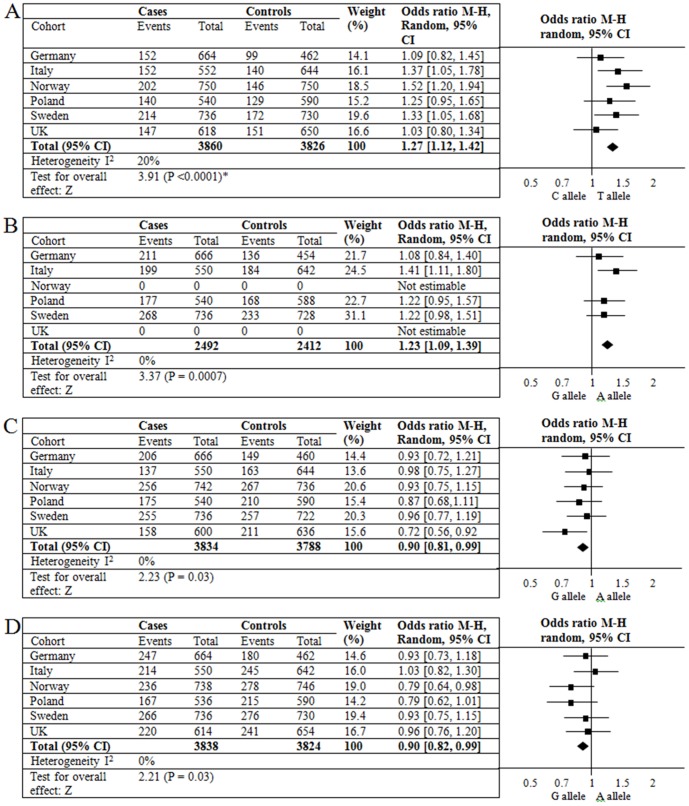
Forest plots of significant meta-analysis results in AAD. Meta-analysis of *rs4274624* and *rs10931481* in the *STAT4* gene (panel A, B), *rs4646536* SNP in the *CYP27B1* gene (panel C) and *rs3802604* SNP in the *GATA3* gene (panel D) in 6 European AAD cohorts. To be included in the analysis, the genotyping call rate per SNP had to be 95% or more for each cohort, in both cases and controls, and the control data set had to not deviate significantly from Hardy Weinberg Equilibrium (P>0.01). Pooled analysis showed little heterogeneity amongst the cohorts (I^2^≤20%). Using a random effects model, the meta-analysis confirms association between alleles at these four SNPs and AAD. Maximum association was noted at *rs4274624* (panel A), with an odds ratio (OR) of 1.27 [95% CI 1.12–1.42), P<0.0001. (*P value = 0.00016 when data analysed under a random effects model in Stata). In panel B, data for the UK and Norwegian cohorts is not presented as the quality control inclusion criteria were not met in these cohorts.

Meta-analysis was performed using genotype data from each of the 6 different patient cohorts. In total, 4 SNPs in 3 genes remained associated with AAD. Maximal association was seen with alleles of two SNPs in moderate LD (r^2^ = 0.59) in the *STAT4* gene (*rs4274624* P = 0.00016; *rs10931481* P = 0.0007) (figure 1). The intronic SNP, *rs4646536*, in *CYP27B1* was also nominally associated in the whole cohort (P = 0.03). This marker is in moderate LD with another genotyped SNP, *rs10876993* (r^2^ = 0.45), however no association was seen with this SNP and AAD (figure 1). Finally, *rs3802604*, an independent SNP in *GATA3*, was also nominally associated with AAD (P = 0.03) (figure 1).

When the meta-analysis was repeated including only 21OH+ AAD individuals (1,204 21OH+ compared to 1,955 individuals in the whole AAD cohort), maximal association was again observed at *rs4274624* in *STAT4* (P = 0.0003). *rs3802604* in *GATA3* also remained nominally associated (P = 0.04). In addition, *rs13017599* in *REL* was associated (P = 0.03) although the result for this SNP in the whole AAD cohort was only marginally significant at P = 0.05. No association was observed with *rs10931481* in *STAT4* (P = 0.07) or with *rs4646536* in *CYP27B1* (P = 0.16).

### Extension of GATA3 analysis

The nominal association of *GATA3* alleles with AAD in UK, Norwegian and Polish cohorts, that remained associated following meta-analysis, was a novel finding (tables 2 and 4), as at the time of this study, polymorphisms in the *GATA3* gene have not previously been associated with autoimmune conditions. This locus was therefore selected for more detailed genotyping in the AAD cohorts and in additional disease cohorts: 1,195 Norwegian subjects with type 1 diabetes, 650 New Zealand subjects with rheumatoid arthritis and 283 UK subjects with Graves' disease.

### GATA3 results

Allowing for 5 independent comparisons at the *GATA3* locus (P 0.05/5 = ≤0.01), 2 SNPs in the UK AAD cohort (*rs569421* P = 0.0096; *rs422628* = P 0.01) remained significantly associated. A further SNP in the New Zealand rheumatoid arthritis cohort (*rs3802604* P = 0.0096) would also meet the significance threshold. *rs3802604* was associated with AAD in the meta-analysis, however in AAD the minor G allele appears to confer protection from disease in contrast to rheumatoid arthritis, where the G allele confers disease susceptibility.

## Discussion

This is the largest study of AAD genetics to date, including almost 2,000 AAD subjects from six European countries. It implicates a number of biomolecular pathways in the pathogenesis of this rare autoimmune condition.

The most robust finding of this study is association of AAD with alleles at two *STAT4* markers. The STAT4 transcription factor is known to have a role in CD4^+^ cell fate, being necessary for generation of T_H_1 responses, and also plays a role in T_H_17 cell differentiation. Variation at the *STAT4* locus is well established as having a role in several different autoimmune conditions including rheumatoid arthritis [24], [25], SLE [23] and primary Sjögren's disease [26]. In the study by Remmers *et al.* [24] the minor allele at *STAT4* marker *rs7574865* was significantly associated with both rheumatoid arthritis (P = 4.64×10^−8^, OR 1.27 [95% CI 1.16–1.36]) and SLE (P = 1.87×10^−9^, OR 1.55 [95% CI 1.34–1.79] in a meta-analysis. The minor allele of this SNP, in addition to three others in intron 3 of *STAT4*, was also associated with rheumatoid arthritis in the Korean population (P = 0.0065, OR 1.27 [95% CI 1.11–1.45]) [25] and with primary Sjögren's syndrome in a small study (P = 0.01, OR 1.47 [95% CI 1.09–1.97]) [26]. The marker most associated with AAD in the meta-analysis performed in this study, *rs4274624*, is in significant LD (r^2^ = 0.90) with SNP *rs7574865*. The other associated marker, *rs10931481*, in the meta-analysis, is in moderate LD with both *rs4274624* (r^2^ = 0.59) and *rs7574865* (r^2^ = 0.53) and was further associated with rheumatoid arthritis and SLE directly in the study by Remmers (P = 0.005, 0.025 respectively) [24], but to a lesser degree than *rs7574865*. These SNPs are all within a large intron in the *STAT4* gene which raises the possibility that they are tagging a variant which, rather than disrupting protein structure and/or function directly as deleterious mutations in the coding regions might, may result in splice variation or disrupt non-coding regulatory components to result in disease susceptibility.

Furthermore, we demonstrated nominally significant association of *GATA3* polymorphisms in UK, Norwegian and Polish AAD populations, and meta-analysis of the whole European AAD cohort further supported this association. GATA3 has been implicated in the homeostasis and regulation of CD8+ T-lymphocytes [27] and could therefore contribute to primary T-lymphocyte dysregulation in autoimmune Addison's disease. Extension of *GATA3* analysis to other autoimmune disease cohorts showed only association with alleles at a single SNP, *rs3802604*, in the New Zealand rheumatoid arthritis population, replicating a recent finding in a large multinational RA patient cohort [28]. This is the SNP that was associated in the AAD cohort meta-analysis, however for AAD, the minor G allele is protective (OR 0.90), whereas in rheumatoid arthritis, the minor G allele confers susceptibility (OR 1.27). Although this may represent a novel but different association in rheumatoid arthritis, reflecting the different immunopathogenesis of this disease compared to AAD, the overall degree of association in AAD is weak and this finding needs further replication in larger datasets.

We have also replicated the association of *CYP27B1* polymorphisms with AAD. Association of a promoter polymorphism in this gene with German autoimmune cohorts, including AAD, Hashimoto's thyroiditis, Graves' disease and type 1 diabetes was first reported in 2004 [29]. This finding was later replicated in small AAD cohorts from the UK and Poland [30], [31]. In this study, we have used meta-analysis to establish an association with an intronic variant in this gene and AAD in European cohorts. There is strong linkage disequilibrium in this region which encompasses the entire *CYP27B1* gene and therefore these associated SNPs may be tagging a more distant causative variant yet to be defined. Alternatively, polymorphisms in *CYP27B1*, also associated with type 1 diabetes [32] may have a role in regulating vitamin D 1-alpha hydroxylation in a tissue-specific manner [33].

Finally, we report significant association with alleles at *NF-κB1* polymorphisms and AAD in the UK cohort and between alleles of an *IL23A* polymorphism and AAD in the Italian cohort. The NF-κB pathway is a highly conserved innate immune mechanism which allows a vigorous and rapid inflammatory response to a myriad of potentially harmful stimuli, and IL23A has a role in CD4^+^ cell fate and the T_H_1 response, therefore these are both plausible candidates for AAD. However, the significance of these isolated findings is currently unclear and these results will need to be confirmed by replication.

We observed significant genetic heterogeneity between the 6 European control cohorts, particularly between geographically distant countries such as the UK and Italy and this may explain the different patterns of associations between the 6 European AAD cohorts. Genetic heterogeneity may also be contributing to the differences observed in the clinical characteristics of the participating European cohorts, for example the age at onset of AAD and the proportion of each cohort with additional autoimmune conditions. However, variation between countries in diagnostic criteria and how these data are recorded and collected is also likely to be a contributing factor to these observed differences. Previous studies have also demonstrated significant genetic heterogeneity between European countries, even when non-Caucasian individuals are excluded. For example, a study by Cross *et al.* published in 2010 [34] compared allele frequencies of 51 SNPs in 19,027 self-reported white Caucasians. Between individuals from Scandinavia, the UK, Germany and Eastern Europe, minor allele frequencies differed significantly (i.e. P<0.05) at 19 (37.3%) SNPs. The difference was particularly marked (i.e. P<0.0001) at 5 (9.8%) of the 51 SNPs analysed. The significant heterogeneity observed between the 6 countries included in this study may account for the different patterns of association in each population and clearly highlights the importance of carefully matching cases with controls in genetic studies.

## Conclusion

This is the largest genetic study of AAD to date and includes almost 2,000 carefully phenotyped individuals from 6 European countries. We have demonstrated significant heterogeneity between control cohorts of the participating European countries. We report that variants in the *STAT4* gene, previously associated with other autoimmune conditions including rheumatoid arthritis and SLE, appear to confer susceptibility to AAD, as demonstrated by data derived from the Italian, Norwegian and Swedish populations studied. We are also able to confirm a nominal association of *GATA3* variants with another autoimmune condition, namely AAD, in UK, Norwegian and Polish Europeans. On further investigation, a single SNP was also associated with rheumatoid arthritis in a cohort from New Zealand, however, the findings could not be replicated in type 1 diabetes or Graves' disease. In addition, we have replicated a previous association with *CYP27B1* polymorphisms with AAD in the UK cohort, with the result supported by meta-analysis in the six European cohorts together. We have found that variants in two genes, *NF-κB1* and *IL23A*, not previously associated with AAD, contribute to susceptibility in the UK and Italian populations respectively, however these findings require replication. Further research, perhaps by genome-wide association studies in large, collaborative cohorts, or whole exome/genome sequencing in selected individuals, is now warranted to determine the genetic factors which make up the remaining hidden heritability of AAD.

## Supporting Information

File S1 **Full genotype data for phases 1, 2 and 3.**
(PDF)
