# Correction: Association of Autoimmune Addison's Disease with Alleles of STAT4 and GATA3 in European Cohorts

**DOI:** 10.1371/journal.pone.0102428

**Published:** 2014-07-03

**Authors:** The PLOS ONE Staff

There is an error in the “Funding” statement. Please refer to the correct “Funding” statement below:

“This work was funded by grants from the Medical Research Council UK to Dr A Mitchell (G0900390) and a European Union Framework 7 grant 201167 to the Euradrenal Consortium.

Financial support for the Swedish contribution was also provided through the regional agreement on medical training and clinical research (ALF) between Stockholm County Council and Karolinska Institutet, The Swedish Society for Medical Research, the Swedish Society of Medicine, the NovoNordisk Foundation, Karolinska Institutet, and the Åke Wiberg Foundation. The funders had no role in study design, data collection and analysis, decision to publish, or preparation of the manuscript.”
