# Supplementary material for: Association of Autoimmune Addison's Disease with Alleles of STAT4 and GATA3 in European Cohorts
Source: PLoS One. 2014 Mar 10;9(3):e88991. doi: 10.1371/journal.pone.0088991 (PMC3948621; doi:10.1371/journal.pone.0088991)
Supplement: File S1 — Full genotype data for phases 1, 2 and 3. (PDF) [file pone.0088991.s001.pdf]

File S1: phase 1, 2 and 3 genotype data

PHASE 1			Norway AAD (n=382)				Norway controls (n=380)						
Gene	RS identifier	minor/ major allele	aa	Aa	AA	Call rate (%)	aa	Aa	AA	Call rate (%)	HWE	P genotype	P allele
CYP2R1	rs11023374	C/T	27	124	205	93	30	112	217	94	0.0065		
	rs1553006	A/G	74	140	109	85	72	111	116	79	0.000031		
	rs10741661	A/G	72	181	121	98	74	181	120	99	0.7	0.98	0.88
TBX21	rs9910408	G/A	76	168	134	99	66	177	130	98	0.67	0.62	0.73
	rs11657388	G/C	12	111	240	95	20	121	230	98	0.44	0.24	0.14
RORC	rs3828057	T/C	74	193	106	98	93	170	112	99	0.078	0.15	0.5
	rs12045886	C/T	27	145	183	93	24	48	196	71	0.000001		
	rs1521177	G/T	80	164	95	89	81	178	112	98	0.52		
	rs7540530	G/A	93	175	106	98	76	188	100	96	0.47	0.33	0.55
	rs949969	A/G	16	122	236	98	14	108	254	99	0.55	0.44	0.22
CYP24A1	rs927650	T/C	89	189	96	98	108	165	102	99	0.02	0.16	0.51
	rs1570669	G/A	45	156	169	97	40	152	172	96	0.46	0.85	0.58
	rs2209314	C/T	17	148	205	97	31	122	217	97	0.024	0.031	0.91
	rs3787555	A/C	23	135	217	98	24	161	189	98	0.18	0.12	0.089
	rs2762941	A/G	39	133	130	79	41	96	150	76	0.00022		
	rs2181874	A/G	11	140	225	98	11	122	239	98	0.33	0.44	0.28
	rs4809959	G/A	76	193	104	98	92	177	106	99	0.29	0.33	0.47
	rs4809960	C/T	15	121	235	97	20	133	213	96	0.9	0.31	0.13
	rs2296241	G/A	84	189	97	97	100	169	102	98	0.087	0.27	0.57
STAT2	rs2066808	G/A	2	51	322	98	0	53	312	96	0.13	0.36	0.96
	rs2066807	G/C	2	53	321	98	0	52	313	96	0.14	0.38	0.73
VDR	rs2189480	T/G	44	173	150	96	58	150	161	97	0.023	0.14	0.85
	rs3819545	G/A	52	176	147	98	60	153	152	96	0.045	0.35	0.98
	rs10875693	A/T	50	123	154	86	41	168	148	94	0.52		
	rs11168275	C/T	22	114	239	98	22	113	232	97	0.11	0.99	0.88
	rs10735810	A/G	63	154	159	98	55	208	112	99	0.0087		
	rs2254210	A/G	69	180	120	97	68	170	120	94	0.57		
IL23A	rs11171806	A/G	2	46	314	95	0	51	319	97	0.15	0.33	0.99
CYP27B1	rs10876994	C/A	28	110	200	88	32	121	189	90	0.021		
	rs4646536	G/A	44	168	159	97	47	173	148	97	0.75	0.76	0.47
	rs703842	G/A	45	172	160	99	46	173	147	96	0.66	0.82	0.56
GATA3	rs3802604	G/A	38	160	171	97	62	154	157	98	0.024	0.04	0.038
	rs570613	C/T	41	183	138	95	62	167	142	98	0.28	0.084	0.3
	rs569421	C/T	9	135	228	97	14	115	233	95	0.97	0.27	0.69
	rs444929	C/T	18	106	241	96	26	121	226	98	0.084	0.24	0.092

**IFIH1**	rs10930046	C/T	0	17	343	94	0	20	334	93	0.58		
	rs13023380	G/A	100	170	105	98	91	176	97	96	53	0.71	0.95
	rs3747517	T/C	26	138	212	98	31	146	193	97	0.65	0.47	0.22
**STAT4**	rs925847	T/C	24	146	195	96	32	126	210	97	0.042	0.21	0.75
	rs3821236	A/G	17	127	229	98	12	110	251	98	0.99	0.21	0.079
	rs16833260	G/C	37	185	144	96	34	163	177	98	0.69	0.09	0.058
	rs3024866	G/A	26	143	197	96	32	127	213	98	0.042	0.34	0.68
	rs1517352	A/C	63	157	142	95	54	164	150	97	0.4	0.6	0.42
	rs10931481	G/A	33	171	133	88	30	152	180	95	0.79		
	rs4274624	C/T	25	152	198	98	9	128	238	99	0.086	0.0013	0.00045
	rs16833215	G/A	36	150	182	96	29	149	192	97	0.99	0.6	0.36
	rs4853543	A/G	0	0	261	68	52	1	308	95	0.000001		
	rs11685878	T/C	65	172	127	95	66	173	125	96	0.65	0.99	0.87
	rs4853546	A/G	48	159	163	97	43	170	161	98	0.85	0.73	0.92
GC	rs4588	T/G	25	133	217	98	26	145	196	97	0.91	0.47	0.29
	rs7041	A/C	70	177	130	99	66	184	124	98	0.87	0.83	0.94
	rs2298849	G/A	15	126	204	90	25	113	227	96	0.04		
**IL17A**	rs4711998	A/G	24	116	231	97	27	142	196	96	0.85	0.06	0.039
	rs3819024	G/A	48	179	144	97	47	162	155	96	0.65	0.55	0.45
	rs16882180	T/C	50	159	151	94	43	163	157	96	0.94		
**IL17RA**	rs2241043	C/T	57	180	137	98	68	163	145	99	0.065	0.36	0.86
	rs6518661	A/G	48	160	168	98	49	162	165	99	0.36	0.98	0.83
**IL21**	rs907715	T/C	41	139	168	91	50	158	157	96	0.31		
	rs2221903	C/T	49	141	149	89	41	108	142	77	0.0078		
**REL**	rs13017599	A/G	47	176	145	96	56	163	151	97	0.28	0.49	0.85
	rs13031237	T/G	9	28	67	27	51	150	142	90	0.27		
**NFATC2**	rs959996	A/G	52	180	142	98	46	182	147	99	0.37	0.79	0.56
	rs2024582	C/A	52	185	135	97	52	180	144	99	0.72	0.85	0.66
	rs6021197	G/T	26	125	224	98	23	130	220	98	0.26	0.86	1
	rs761374	C/T	58	158	154	97	56	153	159	97	0.061	0.91	0.7
	rs2024581	T/C	24	138	202	95	24	106	222	93	0.029		
	rs228832	T/C	23	143	206	97	22	138	215	99	0.1	0.86	0.61
	rs17728712	A/G	27	135	208	97	26	126	223	99	0.16	0.66	0.43
	rs228846	C/T	63	178	128	97	57	137	146	89	0.013		
	rs228851	T/G	85	184	105	98	93	165	93	92	0.26		
	rs6013193	G/T	85	176	111	97	73	165	124	95	0.18	0.4	0.18
	rs6123041	A/T	76	202	96	98	82	176	117	99	0.3	0.13	0.44
	rs3787189	C/T	39	160	175	98	36	161	169	96	0.79	0.93	0.99
	rs2273642	A/G	24	151	197	97	19	137	202	94	0.49	0.59	0.3

	rs6067809	T/C	99	185	89	98	92	194	81	97	0.27	0.67	0.95
**NFKB1**	rs230487	C/A	24	148	197	97	32	155	181	97	0.88	0.37	0.17
	rs10026278	T/C	47	151	176	98	34	158	180	98	0.94	0.32	0.33
	rs230532	T/A	43	137	172	92	33	144	188	96	0.48		
	rs4648072	G/A	0	12	364	98	0	9	355	96	0.81	N/A	0.56
	rs4698861	G/A	40	157	175	97	32	137	187	94	0.34		
	rs228611	A/G	66	189	115	97	91	185	97	98	0.88	0.063	0.024
**RORA**	rs1834336	T/C	59	161	155	98	48	171	148	97	0.9	0.47	0.75
	rs10519085	C/T	109	184	73	96	101	191	80	98	0.56	0.7	0.41
	rs1482058	T/C	98	172	86	93	87	187	89	96	0.56		
	rs1234805	T/C	52	173	145	97	30	160	179	97	0.49	0.0068	0.00187
			UK AAD (n=309)				UK controls (n=335)						
**Gene**	**RS identifier**	**minor/ major allele**	**aa**	**Aa**	**AA**	**Call rate (%)**	**aa**	**Aa**	**AA**	**Call rate (%)**	**HWE**	**P genotype**	**P allele**
**CYP2R1**	rs11023374	C/T	25	107	172	98	25	120	175	96	0.49	0.84	0.77
	rs1553006	A/G	68	136	98	98	67	150	80	89	0.84		
	rs10741661	A/G	66	148	95	100	67	168	93	98	0.58	0.69	0.79
**TBX21**	rs9910408	G/A	58	151	100	100	66	162	96	97	0.88	0.73	0.44
	rs11657388	G/C	22	95	183	97	18	103	198	95	0.35	0.78	0.57
**RORC**	rs3828057	T/C	73	146	90	100	85	161	79	97	0.87	0.38	0.2
	rs12045886	C/T	19	118	142	90	21	106	167	88	0.46		
	rs1521177	G/T	65	142	91	96	72	146	105	96	0.12	0.82	0.8
	rs7540530	G/A	68	141	82	94	85	147	96	98	0.063		
	rs949969	A/G	11	98	197	99	21	98	204	96	0.056	0.25	0.38
**CYP24A1**	rs927650	T/C	81	145	82	100	75	156	99	99	0.37	0.48	0.23
	rs1570669	G/A	41	118	138	96	43	150	133	97	0.94	0.27	0.36
	rs2209314	C/T	13	98	189	97	26	110	176	93	0.14		
	rs3787555	A/C	25	104	176	99	20	132	177	98	0.48	0.23	0.72
	rs2762941	A/G	40	131	126	96	51	140	131	96	0.19	0.7	0.47
	rs2181874	A/G	16	111	182	100	27	116	183	97	0.16	0.29	0.23
	rs4809959	G/A	78	138	93	100	87	175	66	98	0.2	0.012	0.046
	rs4809960	C/T	18	103	183	98	18	116	182	94	0.93		
	rs2296241	G/A	86	143	77	99	56	170	90	94	0.12		
**STAT2**	rs2066808	G/A	0	31	266	96	1	55	269	97	0.3	0.039	0.012
	rs2066807	G/C	0	29	255	92	0	55	270	97	0.26		
**VDR**	rs2189480	T/G	41	151	116	100	42	155	132	98	0.74	0.82	0.57
	rs3819545	G/A	39	149	109	96	57	149	121	98	0.34	0.29	0.47

	rs10875693	A/T	35	139	119	95	32	154	140	97	0.27	0.65	0.4
	rs11168275	C/T	14	132	163	100	17	116	192	97	0.92	0.19	0.23
	rs10735810	A/G	47	139	123	100	53	157	117	98	0.98	0.58	0.36
	rs2254210	A/G	39	151	119	100	46	163	112	96	0.28	0.6	0.32
**IL23A**	rs11171806	A/G	0	30	278	100	0	56	264	96	0.086	N/A	0.0047
**CYP27B1**	rs10876994	C/A	14	89	200	98	19	118	172	92	0.84		
	rs4646536	G/A	25	108	167	97	32	147	139	95	0.45	0.012	0.0091
	rs703842	G/A	26	115	168	100	34	149	142	97	0.58	0.027	0.014
**GATA3**	rs3802604	G/A	39	142	126	99	41	159	127	98	0.42	0.83	0.7
	rs570613	C/T	52	134	118	98	46	152	130	98	0.88	0.56	0.48
	rs569421	C/T	20	112	165	96	9	104	214	98	0.39	0.0092	0.003
	rs444929	C/T	10	108	189	99	26	128	173	98	0.73	0.012	0.0053
**IFIH1**	rs10930046	C/T	0	8	287	95	0	13	302	94	0.71		
	rs13023380	G/A	62	160	75	96	81	160	87	98	0.66	0.39	0.65
	rs3747517	T/C	17	139	152	100	26	133	166	97	0.93	0.34	0.88
**STAT4**	rs925847	T/C	27	108	157	94	35	121	158	94	0.11		
	rs3821236	A/G	14	91	198	98	12	109	193	94	0.48		
	rs16833260	G/C	33	137	138	100	43	139	141	96	0.35	0.6	0.42
	rs3024866	G/A	19	123	166	100	26	127	167	96	0.79	0.56	0.32
	rs1517352	A/C	43	142	114	97	48	143	123	94	0.55		
	rs10931481	G/A	30	137	141	100	38	137	141	94	0.6		
	rs4274624	C/T	17	113	179	100	19	113	193	97	0.65	0.97	0.98
	rs16833215	G/A	32	115	161	100	29	138	162	98	0.96	0.44	0.55
	rs4853543	A/G	50	0	246	96	48	0	267	94	0.000001		
	rs11685878	T/C	46	134	117	96	57	159	99	94	0.62		
	rs4853546	A/G	34	122	150	99	39	142	134	94	0.88		
**GC**	rs4588	T/G	28	126	144	96	31	123	175	98	0.17	0.43	0.36
	rs7041	A/C	57	155	97	100	54	169	101	97	0.24	0.81	0.77
	rs2298849	G/A	6	88	214	100	9	94	211	94	0.7		
**IL17A**	rs4711998	A/G	17	107	174	96	15	138	174	98	0.057	0.26	0.39
	rs3819024	G/A	34	137	127	96	40	158	122	96	0.31	0.52	0.3
	rs16882180	T/C	26	143	133	98	40	166	122	98	0.15	0.13	0.043
**IL17RA**	rs2241043	C/T	42	147	120	100	60	137	132	98	0.023	0.18	0.55
	rs6518661	A/G	36	129	144	100	43	150	134	98	0.92	0.35	0.19
**IL21**	rs907715	T/C	37	122	148	99	47	159	120	97	0.63	0.015	0.012
	rs2221903	C/T	31	114	133	90	23	114	167	91	0.56		
**REL**	rs13017599	A/G	48	155	104	99	30	158	142	99	0.4	0.0099	0.0028
	rs13031237	T/G	44	136	104	92	28	154	140	96	0.11		
**NFATC2**	rs959996	A/G	43	146	117	99	62	146	116	97	0.19	0.23	0.18

	rs2024582	C/A	49	142	114	99	56	145	112	93	0.45		
	rs6021197	G/T	12	115	182	100	19	110	195	97	0.51	0.41	0.88
	rs761374	C/T	47	131	130	100	59	136	130	97	0.029	0.61	0.37
	rs2024581	T/C	20	109	174	98	29	113	168	93	0.12		
	rs228832	T/C	21	108	177	99	21	129	176	97	0.68	0.54	0.49
	rs17728712	A/G	16	118	172	99	24	128	162	94	0.85		
	rs228846	C/T	52	155	101	100	69	154	100	96	0.5	0.36	0.26
	rs228851	T/G	77	150	82	100	73	150	102	97	0.21	0.39	0.2
	rs6013193	G/T	65	124	86	89	85	151	90	97	0.19		
	rs6123041	A/T	77	145	84	99	71	145	100	94	0.19		
	rs3787189	C/T	33	140	136	100	39	121	163	96	0.029	0.13	0.35
	rs2273642	A/G	24	122	150	96	27	121	178	97	0.32	0.57	0.47
	rs6067809	T/C	68	127	102	96	80	163	85	98	0.92	0.065	0.089
**NFKB1**	rs230487	C/A	24	118	164	99	21	136	173	99	0.4	0.67	0.95
	rs10026278	T/C	26	114	169	100	41	142	140	96	0.6	0.012	0.0034
	rs230532	T/A	28	127	149	98	57	146	124	98	0.27	0.0016	0.0004
	rs4648072	G/A	0	4	291	95	0	3	323	97	0.93	N/A	0.61
	rs4698861	G/A	22	125	161	100	45	150	126	96	0.97	0.0002	0.0008
	rs228611	A/G	74	154	80	100	68	147	109	97	0.16	0.11	0.062
**RORA**	rs1834336	T/C	36	125	136	96	41	146	141	98	0.74	0.78	0.56
	rs10519085	C/T	77	145	84	99	95	157	77	98	0.44	0.4	0.18
	rs1482058	T/C	72	151	73	96	79	168	79	97	0.58	0.99	0.95
	rs1234805	T/C	27	137	139	98	37	162	130	98	0.2	0.24	0.093
**PHASE 2**			Germany AAD (n=341)				Germany controls (n=235)						
Gene	RS identifier	minor/ major allele	aa	Aa	AA	Call rate (%)	aa	Aa	AA	Call rate (%)	HWE	P genotype	P allele
**REL**	rs13017599	A/G	45	170	118	98	28	109	95	99	0.7	0.41	0.24
**STAT4**	rs10931481	G/A	33	145	155	98	23	90	114	97	0.41	0.65	0.54
	rs4274624	C/T	15	122	195	97	9	81	141	98	0.53	0.84	0.56
	rs4853543	A/G	40	159	129	96	24	120	83	97	0.044	0.58	0.85
**NFKB1**	rs10026278	T/C	33	133	166	97	24	95	113	99	0.55	0.95	0.76
	rs230532	T/A	34	150	149	98	29	101	102	99	0.61	0.69	0.59
	rs4698861	G/A	29	146	158	98	24	101	106	98	0.99	0.79	0.56
**IL21**	rs907715	T/C	14	133	184	97	25	90	113	97	0.27	0.0078	0.018
	rs2221903	C/T	54	157	122	98	36	93	101	98	0.066	0.2	0.18
**IL17A**	rs3819024	G/A	46	139	147	97	44	89	98	98	0.005		

	rs4711998	A/G	23	128	182	98	13	86	132	98	0.84	0.76	0.47
**GATA3**	rs569421	C/T	25	119	189	98	17	81	133	98	0.34	0.98	0.85
	rs3802604	G/A	52	143	137	97	40	100	91	98	0.17	0.84	0.55
	rs444929	C/T	13	106	213	97	20	69	140	97	0.01	0.06	0.12
**IL23A**	rs11171806	A/G	0	40	293	98	1	39	190	98	0.5	0.12	0.06
**STAT2**	rs2066808	G/A	1	41	291	98	1	40	190	98	0.47	0.27	0.1
	rs2066807	G/C	0	40	293	98	1	39	191	98	0.51	0.12	0.07
**CYP27B1**	rs10876994	C/A	22	122	184	96	16	83	129	97	0.6	0.98	0.97
	**rs4646536**	G/A	34	138	161	98	27	95	108	98	0.39	0.84	0.6
**RORA**	rs1234805	T/C	31	144	157	97	28	95	108	98	0.32	0.55	0.56
**CYP24A1**	rs4809959	G/A	75	165	92	97	58	119	54	98	0.64	0.49	0.26
			Italy AAD (n=280)				Italy controls (n=322)						
Gene	RS identifier	minor/ major allele	aa	Aa	AA	Call rate (%)	aa	Aa	AA	Call rate (%)	HWE	P genotype	P allele
**REL**	rs13017599	A/G	32	138	105	98	36	133	152	100	0.4	0.067	0.082
**STAT4**	rs10931481	G/A	41	117	117	98	26	132	163	100	0.92	0.016	0.0056
	rs4274624	C/T	22	108	146	99	14	112	196	100	0.69	0.059	0.02
	rs4853543	A/G	51	132	89	97	59	154	108	100	0.75	0.97	0.82
**NFKB1**	rs10026278	T/C	27	95	153	98	15	116	191	100	0.62	0.049	0.078
	rs230532	T/A	34	118	124	99	27	130	164	100	0.86	0.17	0.061
	rs4698861	G/A	36	97	142	98	26	112	180	99	0.15	0.13	0.059
**IL21**	rs907715	T/C	34	121	119	98	33	144	145	100	0.75	0.7	0.49
	rs2221903	C/T	21	109	147	99	24	126	171	100	0.91	1	0.95
**IL17A**	rs3819024	G/A	32	124	120	99	37	130	154	100	0.24	0.51	0.4
	rs4711998	A/G	22	98	156	99	22	115	185	100	0.48	0.87	0.68
**GATA3**	rs569421	C/T	17	113	147	99	27	126	169	100	0.61	0.57	0.58
	rs3802604	G/A	38	138	99	98	47	151	123	100	0.95	0.75	0.79
	rs444929	C/T	9	83	186	99	11	69	241	100	0.037	0.063	0.06
**IL23A**	rs11171806	A/G	2	31	241	98	0	18	303	100	0.61	0.012	0.0028
**STAT2**	rs2066808	G/A	3	31	243	99	0	23	298	100	0.51	0.037	0.014
	rs2066807	G/C	2	31	244	99	0	19	297	98	0.58	0.023	0.0063
**CYP27B1**	rs10876994	C/A	10	98	170	99	17	102	202	100	0.39	0.46	0.99

	**rs4646536**	G/A	14	109	152	98	18	127	177	100	0.44	0.96	0.87
**RORA**	rs1234805	T/C	18	95	165	99	18	105	199	100	0.4	0.8	0.49
**CYP24A1**	rs4809959	G/A	80	145	52	99	93	159	68	99	1	0.72	0.69
			Poland AAD (n=275)				Poland controls (n=296)						
Gene	RS identifier	minor/ major allele	aa	Aa	AA	Call rate (%)	aa	Aa	AA	Call rate (%)	HWE	P genotype	P allele
**REL**	rs13017599	A/G	40	119	110	98	47	113	131	98	0.0091		
**STAT4**	rs10931481	G/A	33	111	126	98	25	118	151	99	0.76	0.28	0.13
	rs4274624	C/T	21	98	151	98	18	93	184	100	0.18	0.28	0.11
	rs4853543	A/G	28	124	117	98	36	113	146	100	0.058	0.17	0.45
**NFKB1**	rs10026278	T/C	22	117	129	97	34	139	120	99	0.51	0.16	0.06
	rs230532	T/A	27	112	131	98	37	121	138	100	0.2	0.64	0.43
	rs4698861	G/A	24	110	129	96	33	111	147	98	0.092	0.55	0.89
**IL21**	rs907715	T/C	26	97	147	98	19	114	162	100	0.86	0.35	0.49
	rs2221903	C/T	27	111	128	97	37	147	110	99	0.26	0.04	0.02
**IL17A**	rs3819024	G/A	57	124	89	98	54	150	92	100	0.6	0.5	0.87
	rs4711998	A/G	22	95	153	98	14	102	179	100	0.91	0.23	0.14
**GATA3**	rs569421	C/T	15	89	166	98	18	110	167	100	0.98	0.5	0.28
	rs3802604	G/A	30	107	131	97	37	141	117	100	0.58	0.09	0.06
	rs444929	C/T	13	69	188	98	10	106	180	100	0.24	0.03	0.12
**IL23A**	rs11171806	A/G	2	53	214	98	1	52	241	99	0.3	0.84	0.43
**STAT2**	rs2066808	G/A	2	53	214	98	1	54	241	100	0.26	0.72	0.53
	rs2066807	G/C	2	53	215	98	1	52	243	100	0.31	0.65	0.42
**CYP27B1**	rs10876994	C/A	22	102	145	98	28	120	146	99	0.64	0.58	0.3
	**rs4646536**	G/A	30	115	125	98	39	132	124	100	0.68	0.54	0.26
**RORA**	rs1234805	T/C	47	123	100	98	56	142	97	100	0.75	0.58	0.33
**CYP24A1**	rs4809959	G/A	86	129	55	98	74	143	78	100	0.6	0.11	0.03
			Sweden AAD (n=368)				Sweden controls (n=368)						
Gene	RS identifier	minor/ major allele	aa	Aa	AA	Call rate (%)	aa	Aa	AA	Call rate (%)	HWE	P genotype	P allele
**REL**	rs13017599	A/G	53	180	135	100	50	164	152	99	0.59	0.4	0.27

**STAT4**	rs10931481	G/A	55	158	155	100	41	151	172	99	0.37	0.22	0.08
	rs4274624	C/T	35	144	189	100	21	130	214	99	0.83	0.056	0.017
	rs4853543	A/G	53	177	138	100	53	172	139	99	0.96	0.97	0.92
**NFKB1**	rs10026278	T/C	30	147	191	100	30	169	168	100	0.16	0.22	0.2
	rs230532	T/A	32	152	184	100	29	177	160	99	0.036	0.16	0.25
	rs4698861	G/A	34	160	173	100	31	172	163	99	0.12	0.65	0.71
**IL21**	rs907715	T/C	35	156	177	100	24	151	189	99	0.4	0.29	0.16
	rs2221903	C/T	45	186	137	100	46	165	153	99	0.88	0.35	0.38
**IL17A**	rs3819024	G/A	50	172	146	100	46	172	147	99	0.69	0.92	0.75
	rs4711998	A/G	16	140	212	100	31	135	199	99	0.24	0.07	0.11
**GATA3**	rs569421	C/T	23	132	213	100	20	141	204	99	0.49	0.71	0.79
	rs3802604	G/A	51	164	153	100	59	158	148	99	0.13	0.68	0.51
	rs444929	C/T	16	119	232	100	18	122	225	99	0.78	0.88	0.62
**IL23A**	rs11171806	A/G	1	57	310	100	2	45	317	99	0.77	0.41	0.35
**STAT2**	rs2066808	G/A	1	58	309	100	2	49	316	100	0.95	0.56	0.5
	rs2066807	G/C	1	57	310	100	2	47	317	99	0.86	0.5	0.45
**CYP27B1**	rs10876994	C/A	23	152	193	100	28	171	165	99	0.17	0.31	0.14
	**rs4646536**	G/A	41	173	154	100	38	181	142	98	0.076	0.7	0.7
**RORA**	rs1234805	T/C	43	173	152	100	50	168	148	99	0.83	0.72	0.57
**CYP24A1**	rs4809959	G/A	91	177	100	100	78	173	113	99	0.44	0.4	0.17
PHASE 3			UK AAD (n=335)				UK controls (n=302)						
Gene	RS identifier	minor/ major allele	aa	Aa	AA	Call rate (%)	aa	Aa	AA	Call rate (%)	HWE	P genotype	P allele
GATA3	rs1244181	A/G	10	105	215	99	19	90	188	98	0.074	0.13	0.25
	rs2275806	G/A	43	165	113	96	64	133	97	97	0.15	0.022	0.06
	rs2229359	T/C	0	53	247	90	3	41	256	99	0.2		
	rs3781094	A/C	33	148	147	98	39	120	137	98	0.12	0.34	0.76
	rs3802604	G/A	45	157	132	100	51	119	127	98	0.014	0.17	0.93
	rs570613	C/T	59	146	125	99	53	120	127	99	0.01	0.48	0.4
	rs569421	C/T	21	132	182	100	13	93	194	99	0.66	0.029	0.0096
	rs444929	C/T	11	115	209	100	25	106	169	99	0.16	0.017	0.019
	rs422628	C/T	13	126	188	98	28	111	148	95	0.29	0.013	0.01

	rs406103	T/C	19	125	190	100	11	92	194	98	0.98	0.08	0.026
	rs10905284	C/A	76	147	111	100	68	135	97	99	0.11	0.96	0.88
	rs2229360	T/C	0	2	332	100	0	3	298	100	0.93	N/A	0.57
	rs9746	G/A	10	79	233	96	3	62	230	98	0.6	0.097	0.048
	rs1058240	G/A	11	110	209	99	23	98	178	99	0.073	0.052	0.081
	rs12412241	A/G	26	127	179	99	24	114	161	99	0.45	0.97	0.88
			**UK GD (n=283)**				**UK controls (n=302)**						
**Gene**	**RS identifier**	**minor/ major allele**	**aa**	**Aa**	**AA**	**Call rate (%)**	**aa**	**Aa**	**AA**	**Call rate (%)**	**HWE**	**P genotype**	**P allele**
GATA3	rs1244181	A/G	9	89	176	97	19	90	188	98	0.074	0.22	0.4
	rs2275806	G/A	59	123	97	99	64	133	97	97	0.15	0.96	0.8
	rs2229359	T/C	0	46	224	95	3	41	256	99	0.2	0.15	0.67
	rs3781094	A/C	34	124	122	99	39	120	137	98	0.12	0.77	0.76
	rs3802604	G/A	43	128	111	100	51	119	127	98	0.014	0.43	0.8
	rs570613	C/T	44	142	90	98	53	120	127	99	0.01	0.018	0.17
	rs569421	C/T	17	108	151	98	13	93	194	99	0.66	0.049	0.017
	rs444929	C/T	19	93	162	97	25	106	169	99	0.16	0.73	0.41
	rs422628	C/T	21	104	144	95	28	111	148	95	0.29	0.7	0.47
	rs406103	T/C	17	96	168	99	11	92	194	98	0.98	0.25	0.1
	rs10905284	C/A	60	139	78	98	68	135	97	99	0.11	0.43	0.59
	rs2229360	T/C	0	5	271	98	0	3	298	100	0.93	N/A	0.4
	rs9746	G/A	8	63	181	89	3	62	230	98	0.6		
	rs1058240	G/A	12	95	168	97	23	98	178	99	0.073	0.25	0.33
	rs12412241	A/G	24	111	142	98	24	114	161	99	0.45	0.85	0.61
			**Norway AAD (n=352)**				**Norway controls (n=1353)**						
**Gene**	**RS identifier**	**minor/ major allele**	**aa**	**Aa**	**AA**	**Call rate (%)**	**aa**	**Aa**	**AA**	**Call rate (%)**	**HWE**	**P genotype**	**P allele**
GATA3	rs1244181	A/G	13	103	231	99	44	387	920	100	0.67	0.82	0.54
	rs2275806	G/A	61	159	128	99	220	562	402	88	0.34		
	rs2229359	T/C	0	44	305	99	10	154	1167	98	0.053	0.24	0.82

	rs3781094	A/C	23	141	177	97	132	586	634	100	0.84	0.11	0.042
	rs3802604	G/A	35	151	165	100	157	611	577	99	0.81	0.34	0.15
	rs570613	C/T	36	181	128	98	205	636	510	100	0.77	0.049	0.32
	rs569421	C/T	10	122	217	99	84	462	805	100	0.11	0.05	0.095
	rs444929	C/T	12	113	222	99	66	418	866	100	0.094	0.48	0.71
	rs422628	C/T	14	116	209	96	71	444	831	99	0.25	0.66	0.76
	rs406103	T/C	9	115	226	99	69	430	848	100	0.14	0.13	0.23
	rs10905284	C/A	49	165	133	99	227	642	481	100	0.61	0.41	0.2
	rs2229360	T/C	0	7	340	99	0	45	1265	97	0.53	N/A	0.18
	rs9746	G/A	2	86	259	99	27	311	1004	99	0.61	0.16	0.66
	rs1058240	G/A	12	99	235	98	44	351	786	87	0.54		
	rs12412241	A/G	26	143	179	99	105	546	701	100	0.93	0.96	0.98
			Norway T1DM (n=1195)				Norway controls (n=1353)						
Gene	RS identifier	minor/ major allele	aa	Aa	AA	Call rate (%)	aa	Aa	AA	Call rate (%)	HWE	P genotype	P allele
GATA3	rs1244181	A/G	35	349	808	100	44	387	920	100	0.67	0.86	0.99
	rs2275806	G/A	204	523	315	87	220	562	402	88	0.34		
	rs2229359	T/C	6	137	1044	99	10	154	1167	98	0.053	0.74	0.71
	rs3781094	A/C	124	537	533	100	132	586	634	100	0.84	0.51	0.27
	rs3802604	G/A	154	546	490	100	157	611	577	99	0.81	0.52	0.27
	rs570613	C/T	179	560	451	100	205	636	510	100	0.77	0.99	0.92
	rs569421	C/T	66	421	704	100	84	462	805	100	0.11	0.69	0.93
	rs444929	C/T	49	389	751	99	66	418	866	100	0.094	0.47	0.92
	rs422628	C/T	53	432	704	99	71	444	831	99	0.25	0.17	0.46
	rs406103	T/C	55	388	743	99	69	430	848	100	0.14	0.8	0.94
	rs10905284	C/A	184	612	393	99	227	642	481	100	0.61	0.14	0.65
	rs2229360	T/C	1	61	1127	99	0	45	1265	97	0.53	0.063	0.024
	rs9746	G/A	23	295	873	100	27	311	1004	99	0.61	0.64	0.46
	rs1058240	G/A	41	325	714	90	44	351	786	87	0.54		
	rs12412241	A/G	106	469	617	100	105	546	701	100	0.93	0.57	0.63

			New Zealand RA (n=650)				New Zealand controls (n=452)						
Gene	RS identifier	minor/ major allele	aa	Aa	AA	Call rate (%)	aa	Aa	AA	Call rate (%)	HWE	P genotype	P allele
GATA3	rs1244181	A/G	25	207	414	99	11	150	279	97	0.078	0.4	0.84
	rs2275806	G/A	138	299	196	97	82	204	153	97	0.34	0.29	0.11
	rs2229359	T/C	0	89	557	99	2	62	377	98	0.75	0.23	0.6
	rs3781094	A/C	91	291	255	98	46	197	194	97	0.7	0.13	0.053
	rs3802604	G/A	109	298	233	98	53	201	187	98	0.93	0.033	0.0096
	rs570613	C/T	112	291	241	99	63	200	175	97	0.63	0.39	0.19
	rs569421	C/T	36	237	372	99	13	161	267	98	0.052	0.11	0.13
	rs444929	C/T	39	230	379	100	20	151	270	98	0.85	0.52	0.28
	rs422628	C/T	44	257	343	99	24	159	254	97	0.89	0.26	0.1
	rs406103	T/C	33	221	390	99	12	157	274	98	0.058	0.14	0.3
	rs10905284	C/A	139	311	196	99	76	220	145	98	0.63	0.21	0.12
	rs2229360	T/C	1	19	628	100	0	5	437	98	0.9	0.098	0.026
	rs9746	G/A	20	89	446	85	10	43	293	77	0.000004		
	rs1058240	G/A	33	213	402	100	18	137	287	98	0.75	0.75	0.27
	rs12412241	A/G	49	270	324	99	36	184	220	97	0.78	0.78	0.81
